# Adherence to Healthy and Sustainable Dietary Patterns and Long-Term Chronic Inflammation: Data from the EPIC-Potsdam Cohort

**DOI:** 10.1007/s12603-023-2010-1

**Published:** 2023-10-31

**Authors:** L. Koelman, C. Herpich, K. Norman, F. Jannasch, C. Börnhorst, M.B. Schulze, Krasimira Aleksandrova

**Affiliations:** 1 Department of Molecular Epidemiology, German Institute of Human Nutrition Potsdam-Rehbruecke (DIfE), Nuthetal, Germany; 2 Senior Scientist Group, Nutrition, Immunity and Metabolism, Department of Nutrition and Gerontology, German Institute of Human Nutrition Potsdam-Rehbruecke (DIfE), Nuthetal, Germany; 3 Institute of Nutritional Science, University of Potsdam, Nuthetal, Germany; 4 Department of Geriatrics and Medical Gerontology, Charité Universitätsmedizin Berlin, corporate member of Freie Universität Berlin and Humboldt-Universität zu Berlin, Berlin, Germany; 5 Department of Nutrition and Gerontology, German Institute of Human Nutrition Potsdam-Rehbruecke (DIfE), Nuthetal, Germany; 6 Partner Site Berlin, German Institute for Cardiovascular Research (DZHK), Berlin, Germany; 7 Department of Biometry and Data Management, Leibniz Institute for Prevention Research and Epidemiology (BIPS), Bremen, Germany; 8 Department of Epidemiological Methods and Etiological Research, Leibniz Institute for Prevention Research and Epidemiology, BIPS Achterstr. 30, 28359, Bremen, Germany; 9 Faculty of Human and Health Sciences, University of Bremen, Bremen, Germany

**Keywords:** Chemerin, C-reactive protein, chronic inflammation, EAT-diet, Mediterranean diet, dietary patterns, prospective cohort

## Abstract

**Objectives:**

We explored the prospective associations between adherence to a priori chosen dietary patterns, including EAT-Lancet (EAT-L) and Mediterranean (tMDS) diet with long-term inflammatory responses in a German population sample.

**Design and Setting:**

Prospective cohort study.

**Participants:**

A subsample of 636 predominantly healthy participants from the European Prospective Investigation into Cancer and Nutrition (EPIC)-Potsdam study who were on average 51-years old at baseline.

**Measurements:**

Data was collected repeatedly between 1994/1998–2013. At baseline (1994/1998) and 6.8-years later (2001/2005), EAT-L and tMDS scores were derived from available food frequency questionnaires. Stable high, stable low, increasing, and decreasing adherence to EAT-L and tMDS were defined as scoring above/below baseline median at baseline and 6.8-years later. Long-term chronic inflammation was assessed based on the average values of repeated measurements of two inflammatory biomarkers - chemerin and high-sensitivity C-reactive protein (hs-CRP) - in plasma samples collected between 2010/2012 and 2013. Multivariable linear regression analysis adjusted for socio-demographic and lifestyle factors at baseline and in 2010/2012 was used to assess the association between diet adherence and long-term hs-CRP and chemerin concentrations.

**Results:**

Stable high or increasing adherence to EAT-L diet compared to stable low adherence was associated with slight reduction of long-term chemerin concentrations on the long run (stable high: −4.4%; increasing: −4.0%), not reaching statistical significance. Increasing adherence to tMDS compared to stable low adherence was also associated with a minor reduction in chemerin concentrations (−3.6%). Decreasing adherence to tMDS compared stable high adherence was associated with 2.7% higher chemerin. The associations were even less pronounced when hs-CRP was used as an outcome.

**Conclusions:**

Adherence to healthy and sustainable dietary patterns defined using existing definitions for EAT-L and tMDS were associated with minor and not statistically significant reduction in the concentrations of inflammatory biomarkers on the long run. More research is needed to explore whether following these diets may represent a suitable approach for targeted prevention in the general population.

## Background

Unresolved inflammatory response, known as chronic inflammation, is suggested to play a central role in the early-stage pathophysiology of major chronic diseases (1), and may also have a strong implication in the progression and severity of infections, including SARS-CoV-2 (2). Strengthening the immune system is therefore believed to play an important role in countering pro-inflammatory processes and determining disease outcomes (3). Healthy lifestyle has been regarded as an important first-line approach to supporting the immune system in the prevention and treatment of disease risk (4). The extent to which lifestyle factors, including diet, might influence immune response and modulate systemic chronic inflammation on the long run remains unclear. So far, several randomized controlled trials (RCTs) reported on the association between dietary patterns and inflammatory biomarkers (5, 6, 7, 8). Overall, these trials could demonstrate that short-term adherence to the Mediterranean diet (tMDS) was associated with lower levels of inflammatory biomarkers, such as C-reactive protein (CRP) and interleukin-6 (IL-6) in obese and metabolically dysfunctional participants. However, the association between dietary patterns and long-term inflammatory response in the general population has not been sufficiently explored (7, 9). More specifically, evidence on the role of emerging dietary patterns, such as the planetary health diet (EAT-Lancet (EAT-L)) in relation to inflammatory status is limited (10). Furthermore, while most previous studies have focused on established biomarkers of inflammation such as CRP, evidence on novel inflammatory biomarkers has been scant. Among a set of novel candidate biomarkers, recent research has suggested chemerin as a promising inflammatory biomarker linked to a plethora of chronic diseases, incl. cardiovascular disease (CVD), type2 diabetes and cancer (11, 12, 13, 14). Chemerin is known as a pleiotropic chemokine and adipokine with multiple functions. Its adipose tissue expression and serum levels are increased in obese individuals and it plays a role in adipogenesis, glucose homeostasis (15), and immune cell trafficking (16). We previously reported that chemerin was associated with dietary factors (17), and chemerin was reduced after patients with morbid obesity and type 2 diabetes followed different types of plant and animal-based intervention diets (18, 19). So far, no studies have been conducted in community dwelling individuals. To address these gaps, the present study aimed to explore the prospective association between EAT-L and tMDS scores and changes in these scores over a 6.8-year period with chronic inflammatory state 8.6-years later assessed by average concentrations of two inflammatory biomarkers - chemerin, as novel and specific biomarker of immune activity and inflammageing, and hs-CRP as established inflammatory biomarker - measured repeatedly 2–3 years apart.

## Methods

### Study population and data collection

The sample was drawn from a sub-study of the European Prospective Investigation into Cancer and Nutrition (EPIC) - Potsdam cohort. EPIC - Potsdam is a prospective cohort study intended to investigate the role of diet in the development of chronic diseases (20). In the period of 1994/1998, 27548 individuals from Potsdam, Germany and the surrounding geographical area were recruited. Detailed information about recruitment procedures have been reported elsewhere (21). At baseline (T0), information on socio-economic and demographic characteristics, lifestyle factors, i.e., smoking and physical activity, and medical history were collected using computer-guided interviews. In physical examinations at the study center, anthropometric [body height, body weight, waist circumference (WC)] measurements were conducted according to standardized protocols as previously reported (20), and blood was drawn. Habitual dietary intakes of 12 months prior to recruitment were assessed through validated 148-item semi-quantitative food frequency questionnaires (FFQs) (22). In 2001/2005 (T1), 24200 participants were asked to complete another FFQ at study follow-up. During 2010/2012 (T2), a subsample of 815 participants was randomly selected from 23881 cohort members that were still actively participating in the follow-up. According to a rectangular sampling scheme, equal representation of men and women, and equal participants in each of the three 10-year categories for age at baseline (35–44 years, 45–54 years, 55–64 years) were selected. Physical examinations including blood sampling as well as inquiries on lifestyle were conducted. In 2013 (T3), another follow-up assessment was performed involving the 815 individuals that included blood sampling.

**Figure 1:**
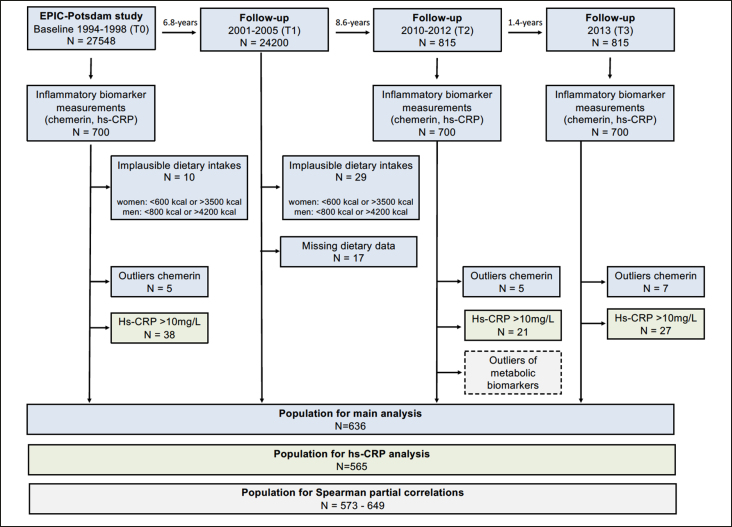
Flow chart of study population included in the EPIC-Potsdam sub-study

For the current study, 700 participants with repeated biomarker measurements were initially included in the analysis (see Figure 1 for flow-chart). Participants were excluded if they had missing values of energy intake and implausible dietary intakes at T0 or T1 (women: <600 kcal or >3500 kcal; men: <800 kcal or >4200 kcal) or had chemerin concentrations above the 99th percentile (n=17). The analytical sample with chemerin as outcome included 636 participants. The analytical sample for hs-CRP included 565 participants after exclusion of participants with probable acute inflammation based on CRP values above 10 mg/L (see Figure 1).

### Dietary patterns

We derived a priori EAT-L diet scores and tMDS scores at T0 and 6.8-years later (T1). These patterns have been previously investigated in relation to health outcomes [EAT-L (22, 24, 25); tMDS (26, 27, 28)]. The EAT-L diet score was constructed based on the 14 dietary recommendations in the EAT-Lancet report (10). Participants were assigned proportional scores ranging from 0–10 for each recommendation, that were summed resulting in a score ranging between 0 (no adherence) and 140 (complete adherence). The tMDS was adapted from Trichopoulou (26, 29), and scoring was based on tertile intake levels of nine key components, with a maximum of 18 points (complete adherence). In our study, the EAT-L score ranged from 17–83 points, whereas the tMDS ranged from 1–16 points. More details on pattern score generation is presented in Supplementary Material [see Supplementary Tables 1–3].

### Biomarker measurements

Participants provided 30 mL peripheral venous blood at T0, T2 and T3, which was processed and subsequently stored in tanks of liquid nitrogen at −196°C or in deep freezers at −80°C until time of analysis. Chemerin and hs-CRP were measured in citrate-treated plasma at T0 and in EDTA plasma at T2 and T3 with a commercially available sandwich enzyme-linked immunosorbent assay (ELISA) (BioVendor, Brno, Czech Republic) at the department of Nutrition and Gerontology at DIfE (Nuthetal, Germany). Citrate measurements at baseline were corrected for the dilution to improve comparability with concentrations measured in EDTA-plasma (30). Coefficients of variation of chemerin reported by the manufacturer were 5.1% and 7.0% within assays and 6.9% and 8.3% between assays. Chemerin measurements showed good reproducibility over 4-months [intraclass correlation coefficient: 0.72 (95%-CI: 0.65–0.78)], indicating that one-time chemerin measurements are reasonably representative of the average individual concentration over time (31). Long-term inflammation was assessed as the average chemerin and hs-CRP concentrations measured at T2 and T3.

At T2, cortisol concentrations were determined from hair strands using CLI (commercially available immunoassay with chemiluminescence detection (CLIA), IBL-Hamburg, Germany), as previously described (32). The within and between-assay variability were below 8% (32). Albumin was determined in serum using colorimetry at a central laboratory (IMD Labor, Potsdam, Germany). Low-density lipoprotein cholesterol (LDL-c) was estimated in serum by photometric technology (IMD Labor, Potsdam). Creatinine, high-density lipoprotein cholesterol (HDL-c) and triglycerides were estimated in serum using ELISA (IMD Labor, Potsdam). Glycated hemoglobin (HbA1c) was measured in EDTA-blood using high performance liquid chromatography (IMD Labor, Potsdam).

### Covariates assessed at baseline and T2

In multivariable-adjusted analyses, model was adjusted for participants' baseline characteristics (sex, age, body mass index (BMI), waist circumference (residually adjusted for BMI to reduce collinearity between these two variables), recreational sports (hours/week), educational attainment (no training/vocational training, technical college, university), smoking status (never, former, ever), and prevalent diseases [hypertension (+ antihypertensive medication), type 2 diabetes, cancer (except non-melanoma skin cancer), CVD]), and baseline corresponding chemerin or hs-CRP concentration. The analyses were further adjusted for total energy intake (kcal/day) at baseline and T1, and available covariates at T2, including smoking status, recreational sports, alcohol intake (EAT-L analysis), and prevalent diseases [hypertension, type 2 diabetes, cancer, CVD]. The analyses modeling EAT-L as main exposure were additionally adjusted for alcohol intake (0, >0–6, >6–12, >12–24, >24–60, >60–96 g/d) to allow comparability with previous publications (29) and tMDS.

### Statistical analysis

Chemerin and hs-CRP were log-transformed to normalize its distribution. The EAT-L diet score and tMDS were dichotomized using the following median cut-off values for EAT-L (low <52.7 / high ≥52.7 points) and tMDS (low <8 / high ≥8 points) at T0, and upper quartile cut-off values for EAT-L (low <60.1 / high ≥60.1 points) and tMDS (low <10 / high ≥10 points) at T0. Correlations between chemerin concentrations with hs-CRP, anthropometry, age, and metabolic biomarkers measured in blood samples collected in 2010/2012 (T2) were evaluated using Spearman partial correlation analyses adjusted for age and sex. To evaluate whether a change in adherence to EAT-L and tMDS over 6.8-years was associated with inflammatory biomarkers 8.6-years later, we derived a variable with four categories for each diet score: stable high score (participants who maintained a high score at T0 and T1), stable low score (participants who maintained a low score at T0 and T1), increasing score (participants who increased their score from T0 to T1) and decreasing score (participants who decreased their score from T0 to T1). We conducted two types of analysis in parallel: for the main analysis, cut-off values of high/low score adherence was based on the median baseline, to be consistent with the previous study by Akbaraley et al. (33). We also included analysis for cut-off values based on upper quartile vs lower quartile. Adjusted geometric means of long-term chemerin and hs-CRP were calculated for all four dietary pattern adherence categories. The percentage differences were calculated for the following categories: stable high vs stable low score, increasing vs stable low score, and decreasing vs stable high score following a similar approach to Akbaraley et al. (33) using the formula: (exp(regression coefficient)-1)*100.

### Stratified analyses

Stratified analyses for chemerin were performed to determine whether results were affected by participants according to baseline age (<50.8 years / ≥50.8 years), sex (men / women), weight (BMI <25 kg/m^2^ / ≥25 kg/m^2^) and weight change from T0 to T2 (cut-points for weight loss, weight maintained, weight gain based on Paige et al. (2014) (34)).

Missing values of all covariates were imputed with random forest using the R package missForest (35). Statistical analyses were performed in SAS (version 9.4, Enterprise Guide 7.1, SAS Institute Inc., Cary, NC, USA) and R (version 3.4.3, R Foundation for Statistical Computing, Vienna, Austria) in R studio (version 0.99.903, RStudio Inc., Boston, MA, USA).

## Results

The baseline characteristics of the study participants are presented in Table 1, overall and by sex. The mean (SD) age of the study participants was 50.8 (8.1)-years and BMI was 26.3 (3.7) kg/m^2^. Median (IQR) baseline EAT-L score was 52.7 (44.4, 60.1) and tMDS was 8.0 (7.0, 10.0). The proportion of high adherence to EAT-L and tMDS scores at baseline were 49.8% and 63.1%, respectively. Overall, men were more likely to have a higher education degree, a full-time job, hypertension, be a smoker, be higher alcohol consumers, and have higher energy intake. Women had higher adherence to EAT-L diet score than men [56.4% vs 43.8%, respectively] and higher concentrations of hs-CRP [median (IQR) 2.21 (0.95, 3.64) vs 1.67 (0.66, 3.00) mg/L, respectively]. Average follow-up time from T0-T1, T1-T2, and T2-T3 was 6.8-years, 8.6-years, and 1.4 years, respectively.

**Table 1:** Baseline characteristics of study population, overall and by sex

		**Total N=636**	**Men N=333**	**Women N=303**
Age	Age [years], mean (SD)	50.8 (8.1)	51.9 (7.7)	49.7 (8.4)
Education	University degree, n (%)	291 (45.8)	186 (55.9)	105 (34.7)
Employment	Full time [≥35 h/week], n (%)	460 (72.3)	259 (77.8)	201 (66.3)
Anthropometry	Body mass index [kg/m^2^], mean (SD)	26.3 (3.7)	27.0 (3.4)	25.6 (3.9)
	Waist circumference [cm], mean (SD)	87.4 (12.0)	94.3 (9.7)	80.0 (9.7)
Blood pressure	Systolic [mmHg], mean (SD)	129.5 (16.5)	132.8 (15.5)	125.9 (17.0)
	Diastolic [mmHg], mean (SD)	84.0 (9.8)	85.8 (9.6)	82.0 (9.6)
Prevalent condition	Hypertension, n (%)	290 (45.6)	169 (50.8)	121 (39.9)
	Cardiovascular disease, n (%)	14 (2.2)	9 (2.7)	5 (1.7)
	Type 2 diabetes, n (%)	19 (3.0)	11 (3.3)	8 (2.6)
	Cancer, n (%)	20 (3.1)	8 (2.4)	12 (4.0)
Medication use	Lipid-lowering, n (%)	37 (5.8)	20 (6.0)	17 (5.6)
	Aspirin, n (%)	2 (0.3)	1 (0.3)	1 (0.3)
	Anti-diabetic, n (%)	9 (1.4)	6(1.8)	3 (1.0)
	Anti-hypertension, n (%)	106 (16.7)	60 (18.0)	46 (15.2)
Physical activity	Recreational sports [h/w], median (IQR)	0.0 (0.0, 2.0)	0.0 (0.0, 2.0)	0.0 (0.0, 1.0)
Smoking	Current smoker, n (%)	103 (16.2)	64 (19.2)	39 (12.9)
Alcohol consumption	Intake of alcohol consumers [g/d], median (IQR)	9.9 (4.4, 20.8)	16.1 (8.2, 26.0)	6.0 (2.4, 12.0)
Energy intake	Kcal, mean (SD)	2127.9 (629.9)	2388.7 (638.7)	1841.3 (479.2)
Inflammatory biomarkers	Chemerin [ng/mL], median (IQR)	229.1 (192.9, 272.4)	227.2 (193.3, 273.0)	230.6 (192.5, 271.7)
	hs-CRP [mg/L], median (IQR)*	1.86 (0.78, 3.31)	1.67 (0.66, 3.00)	2.21 (0.95, 3.64)
Dietary patterns	EAT-L score, median (IQR)	52.7 (44.4, 60.1)	50.6 (43.1, 57.8)	55.4 (45.9, 62.4)
	High adherence EAT-L score, n (%)	317 (49.8)	146 (43.8)	171 (56.4)
	tMDS, median (IQR)	8.0 (7.0, 10.0)	8.0 (7.0, 10.0)	8.0
	High adherence tMDS, n (%)	401 (63.1)	200 (60.1)	201 (66.3)


In subsample with outliers and >10 mg/L removed (Total N=563; Men N=299; Women N=264; Abbreviations: CRP, C-reactive protein; EAT-L, EAT-Lancet Planetary Health Diet; hs, high sensitivity; tMDS, traditional Mediterranean Diet Score

The Spearman partial correlations at T2 between chemerin, hs-CRP, anthropometric parameters, age, and metabolic biomarkers are presented in Table 2. Chemerin showed strong positive correlations with hs-CRP, BMI, waist circumference, triglycerides, creatinine, and an inverse correlation with HDL (P<.0001).

**Table 2:** Spearman partial correlation coefficients and 95% CIs for chemerin and hs-CRP, BMI, waist circumference, age, and various blood-based biomarkers all measured at T2^a^, adjusted for age and sex

		**Chemerin**		
		**N**	**rho**^b^	**95%-CI**^c^
Inflammation	hs-CRP	625	0.24	0.16, 0.31
	P-value	<.0001		
Anthropometry	BMI	649	0.24	0.16, 0.31
	P-value	<.0001		
	Waist Circumference	649	0.26	0.18, 0.33
	P-value	<.0001		
Age	Aged	649	0.10	0.02, 0.18
	P-value	0.01		
Liver metabolism	Albumin	643	0.11	0.03, 0.19
	P-value	0.005		
Kidney function	Creatinine	645	0.18	0.10, 0.25
	P-value	<.0001		
Glucose metabolism	HbA1c	645	0.10	0.02, 0.18
	P-value	0.011		
Lipid metabolism	HDL	645	−0.16	−0.23, -0.08
	P-value	<.0001		
	LDL	645	0.07	−0.01, 0.14
	P-value	0.093		
	Triglycerides	645	0.22	0.14, 0.29
	P-value	<.0001		
Chronic stress	Cortisol	573	0.02	−0.06, 0.11
	P-value	0.554		


a. The sub-sample for Spearman partial correlation analyses ranged between N=573 - 649, depending on the biomarker and respective outliers; b. Spearman partial correlation coefficient; c. Based on Fisher's z transformation; dAdjusted for sex only; Abbreviations: BMI, body mass index; CI, confidence interval; HbA1c, glycated hemoglobin; HDL, high-density lipoprotein; hs-CRP, high sensitivity C-reactive protein; LDL, low-density lipoprotein

**Figure 2:**
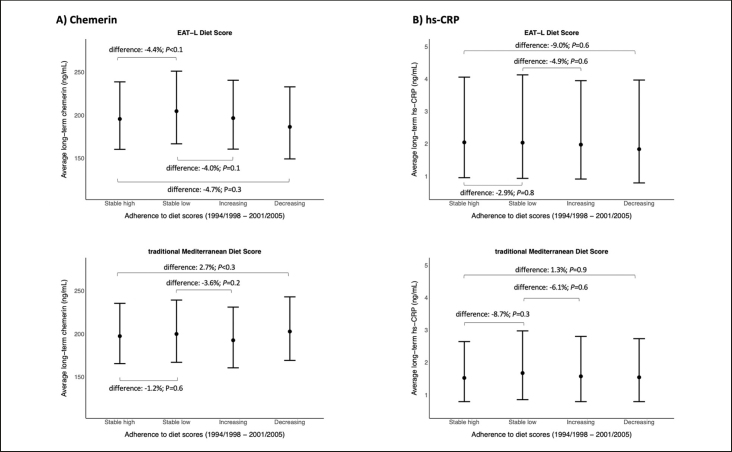
Adjusted geometric means of A) chemerin and B) hs-CRP from the average of two blood samples drawn between 2010–2013 according to changes in dietary pattern score adherence

Figure 2 presents the geometric means and percentage differences of long-term chemerin and hs-CRP by categories of diet score adherence over 6.8-years. Those who maintained a low EAT-L score had highest chemerin and hs-CRP concentrations, and those with decreasing EAT-L score had lowest chemerin and hs-CRP concentrations. For the tMDS, highest average chemerin and hs-CRP were found in those with decreasing and lowest score adherence, respectively. Vice versa, lowest chemerin and hs-CRP were observed in those with increasing and highest score adherence, respectively. Compared to stable low adherence to EAT-L, a stable high or increasing adherence showed lower long-term chemerin (4.4% lower, P<0.1 and 4.0% lower, P=0.1, respectively). Compared to stable low adherence to tMDS, increasing adherence showed a decreasing trend with chemerin (3.6% lower, P<0.2). Vice versa, compared to stable high adherence to tMDS, decreasing adherence showed an increasing trend with chemerin (2.7% higher, P<0.3). For hs-CRP as outcome, stable high or increasing adherence to tMDS was weakly associated with lower long-term hs-CRP compared to stable low adherence (8.7% lower, P=0.3 and 6.1% lower, P=0.6, respectively). These results did not reach statistical significance.

Change in dietary pattern adherence over the exposure period (1994/1998 to 2001/2005) included stable high, stable low, increasing, and decreasing score based on the median baseline scores; Model adjusted for baseline age, sex, education, anti-hypertensive medication, BMI, waist circumference residual, chemerin (A) or hs-CRP (B), smoking status at baseline and T2, recreational sports at baseline and T2, alcohol intake (EAT-L analysis at baseline and T2), prevalent diseases at baseline and T2 (cancer, diabetes, cardiovascular disease, hypertension), total energy intake at baseline and T1

Different adjustment models did not alter results for chemerin substantially [see Supplementary Table 4]. Repeating analyses with diet adherence cut-off based on upper quartile (instead of median) also revealed weaker associations [see Supplementary Table 5].

### Stratified analyses

Stratified analyses by age revealed that those of older age had increasing adherence to EAT-L (36% vs 27%). In older aged individuals, stable high and increased EAT-L compared to low adherence were more strongly associated with lower chemerin. Stratified analyses by sex revealed that associations between diet score adherence and changes in chemerin were stronger in men compared to women. Over time, 35% of men increased their adherence to EAT-L, in women this was 28%. In men associations were strongest for stable high EAT-L and tMDS scores in reducing chemerin concentrations (−6.8% difference; P=0.05). Decreasing tMDS score compared to maintaining high score was associated with 7.1% higher chemerin (P=0.06).

Weight change from T0 – T2 was positively associated with long-term concentrations [Spearman partial correlations adjusted for age and sex (rho: 0.12; 95%-CI: 0.04, 0.19; P<0.01)]. Most participants gained weight from baseline to T2 (59% with a mean (SD) of 2.7 (7.0) kg). In those who gained weight, chemerin concentrations were higher compared to those who maintained or lost weight [see Supplementary Table 6]. When adding weight change categories to the adjustment model, associations attenuated towards null [see Supplementary Table 7]. When stratifying for baseline BMI, associations between stable high vs low EAT-L score and reduced chemerin were only seen in participants with BMI below 25 kg/m2 (9.5% lower, P<0.02).

## Discussion

In this prospective cohort study, we analyzed the association between adherence to EAT-L and tMDS diets over 6.8-years with long-term inflammatory levels measured based on chemerin and hs-CRP concentrations. Compared to stable low adherence, high adherence to EAT-L and increasing adherence to EAT-L and tMDS was associated with overall minor albeit not statistically significant reductions of chemerin and hsCRP concentrations on the long run. Associations were more pronounced in men, individuals of older-age, and in individuals that were normal weight at study baseline. Weight change seemed to be the most prominent factor influencing the observed associations.

So far, the influence of diet on inflammation in the general population has been investigated by a number of observational studies. Among these, cross-sectional studies have abundantly reported on the association between higher anti-inflammatory diet scores (mostly Mediterranean diet) and lower inflammatory markers (7, 9). However, evidence from longitudinal studies on association between long-term adherence to a healthy diet with subsequent chronic inflammation in a general population has been limited with the majority of studies reporting no association (7). Furthermore, intervention studies have mostly shown a positive effect of short-term adherence to anti-inflammatory diets in individuals with underlying clinical metabolic condition, whereas in nonclinical and healthy populations, no meaningful reduction in inflammatory biomarker levels has been observed (36). Our findings showing only minor and not statistically significant reduction in the levels of both chemerin and CRP in the long run add to this evidence suggesting that in the general population adherence to EAT-L and tMDS diets may not be effective in reducing inflammatory levels. However, the results may also reflect the methodological challenges in depicting both changes in diet and inflammatory levels over time in healthy populations, such as the short-term nature of the inflammatory response and variability in dietary patterns. Thus, we hypothesized that changes in diet may influence inflammatory status later on in life, however, we could not account for any changes in between the last dietary measurement and chemerin (8.6 years). As people age, habitual diet and other behaviors may continue to change over time, so the weak and insignificant longitudinal associations found may be due to short-term variability in diet. Another possibility may be that the effect of investigated dietary patterns in the entire study sample have been diluted due to between-person variability and the results may not have sufficiently depicted the possible role of following these diets in specific population subgroups, as seen in the stratified analyses by sex and obesity status. Finally, a priori defined dietary patterns, based on known healthy diet scores, have been shown to be less likely associated with inflammation compared to patterns derived using data-driven methods which are more suitable in explaining variation within the data (7). Further work is therefore warranted to evaluate empirically derived diet scores explaining variation in long-term concentrations of inflammatory biomarkers.

To our knowledge, this is the first study to evaluate the association between EAT-L diet score and inflammatory biomarkers. So far, adherence to EAT-L was consistently associated with lower incidence of non-communicable chronic diseases (23, 24, 37). The EAT-L diet has common features with the Mediterranean diet, being largely plant-based with high intakes of fruits, vegetables and nuts, and low intakes of animal protein such as red meat and dairy. A growing body of evidence suggests that several pro-inflammatory biomarkers including CRP concentrations are reduced in people following the Mediterranean diet (6, 8). The mechanisms through which plant-based diets may decrease circulating levels of biomarkers remain unclear, but individual components as well as their synergy seem to possess anti-inflammatory and immune-balancing properties (8). We previously reported that chemerin concentrations were decreased after a 6-week intervention with diets high in animal (AP) or plant (PP) protein foods (18), with a larger decrease seen in the AP group. The AP diet consisted mainly of dairy products, lean meat, fish and eggs, whereas the main sources of protein in the plant diet were legumes, fruits and pea-protein rich foods. In previous work based on cross-sectional population-based sample, we observed linear associations between elevated chemerin with higher intakes of sugar sweetened beverages and red meat, and lower intakes of dairy products, fish and whole grains (17). Thus, specific foods including dairy, fish, and whole grains seem to be associated with lowering circulating chemerin. Our results add to the limited body of evidence on complex dietary patterns in relation to chemerin.

CRP is a biomarker that is most widely accepted and often used as proxy indicator of chronic inflammation. However, its concentrations may not reflect specific immune-inflammatory responses for chronic disease etiology (38). So far, one cross-sectional study reported on associations of several data driven dietary patterns on biomarkers including chemerin (39). The healthy pattern (consisting of i.e., vegetables, fruits, fish, nuts, and seeds) and dairy pattern (i.e., cheese, egg, milk, fermented milk, fiber-rich breads, nuts, and seeds) showed inverse associations with chemerin, whilst the Western/traditional and fast-food pattern were positively associated with chemerin. Our study on long-term adherence to Mediterranean-type diets on chemerin point to a weaker association. Similar to results from the prospective Whitehall II Study (33), we found a trend of deleterious effects of low adherence to healthy dietary patterns, as decreasing adherence to tMDS compared to maintaining high adherence was associated with higher levels of chemerin in men. Not only healthy dietary behavior influences long-term chronic inflammation but moving towards unhealthy patterns may have unfavorable effects over time.

We reported seemingly higher levels of chemerin in our study sample compared to others (31, 40, 41), which may be explained by population characteristics, i.e., older-aged individuals with high prevalence of hypertension and impaired metabolic health. Women had higher adherence to EAT-L and tMDS at baseline, whilst men increased their adherence over time. These findings come in support of a recent report detailing dietary trends world-wide (42). Based on data from the Global Dietary Database, high income countries have increased their consumption of healthy foods between 1990 and 2018. Women also seem to have higher adherence to healthy diet indices compared to men (42). Individuals in our sample were well educated, and education had a significant impact on the consumption of healthier foods in Central Europe (42).

The associations between healthy diets and chemerin were stronger in men. Sex-differences may exist in the regulation of systemic inflammation, and sex hormones likely contribute to the synthesis of chemerin (43). In our previous work (17) and confirmed among the literature (44, 45), chemerin concentrations are higher in women than men (43), yet limited data exists on differences on the impact of diet. Results from a controlled feeding study suggests that the variability in the anti-inflammatory effects of the tMDS may be attributed to the individuals overall inflammatory status in men (46). An observational study reported reduced CRP in men with high tMDS adherence (47), while another observed this association irrespective of sex (48). Further research is needed in understanding mechanistic differences within a population, in order to strengthen population guidance and allow for more precise dietary requirements.

In addition to diet, other lifestyle modifications such weight loss and exercise have shown to influence circulating chemerin (17). In participants with obesity following a 12-week weight-reducing dietary intervention, the reduction in chemerin concentration was associated with a change in BMI (49). These results are further supported by a study in rats that were fed a calorie restricted diet, and serum chemerin as well as messenger ribonucleic acid expression in adipose tissue were reduced (50). Upon refeeding, serum and mRNA concentrations of chemerin in white adipose tissue were reported. No changes in chemerin mRNA levels in the liver were observed, suggesting that adipose tissue may be an important dietary modifiable source of chemerin (50). Indeed, the association of chemerin with obesity is well-established (51), confirming the positive associations we found of chemerin with BMI, waist circumference, and weight gain. As an adipokine that is predominantly expressed in adipocytes within white adipose tissue, the secretion of chemerin increases with adipocyte differentiation and obesity (52). We saw that individuals who lost weight had higher adherence to EAT-L diet and lower long-term chemerin compared to individuals who gained weight. We also saw that those who maintained their weight had similarly low chemerin concentrations. Some studies have highlighted that at least 10% weight loss is needed to have an effect on inflammatory biomarkers in individuals with overweight and obesity (53). In our study, the associations of high EAT-L adherence were stronger in normal weight individuals at study baseline as compared to overweight or obese, indicating effect modification by weight status.

This study has several strengths. The longitudinal study design made it possible to detect long-term changes in exposure over time in relation to the outcome. This is a major advancement as it enables us to learn more about the temporality of the association and rule out reverse causation. We took into account changes in lifestyle factors in our model, as these are bound to happen over time. We also constructed and evaluated a detailed version of the EAT-L diet score - a promising plant-forward diet that was proposed by leading researchers within the field and can be put together with foods from cultures on a global scale.

The present study is also prone to limitations. The EPIC-Potsdam sample is not a representative sample of the general population. Participants are more health-conscious, have a better health status, and higher socio-economic status than the general population in the east of Germany (21).

We did not take locally grown foods into account when constructing the EAT-L diet score as this data was not available, yet an important aspect to be considered when determining sustainability of food (54). Nevertheless, the FFQs were based on foods in Germany, so some aspects of locally available foods may have been accounted for. Also, the FFQs were applied in the' 90s, so they may not reflect the intake behavior of participants nowadays. The adherence to diet scores were dichotomized, so information on smaller changes in adherence may not have been sufficiently represented. A methodological limitation of this study is that there were differences in the FFQs at study baseline and follow-up. Despite correcting for estimated differences of intake in major food groups, measurement error cannot be ruled out. Another drawback is that not all covariates were measured repeatedly at each time point. We adjusted for changes in lifestyle factors over time, but the results may be affected by time-dependent confounding. As we used repeated data that was available, the measurement schedule of this study was arbitrary. There was no biological reason behind the amount of time between the follow-up measurements, i.e., the time between changes in diet and long-term chemerin concentrations were not specifically established for this study.

In conclusion, adherence to healthy and sustainable dietary patterns defined using existing definitions for EAT-L and tMDS was associated with a minor and not statistically significant reduction in the concentrations of inflammatory biomarkers on the long run. More research is needed to explore whether following these diets may represent a suitable approach for targeted prevention in the general population.

## Electronic Supplementary Material

Supplementary material, approximately 52 KB.
