# Supplementary material for: Adherence to Healthy and Sustainable Dietary Patterns and Long-Term Chronic Inflammation: Data from the EPIC-Potsdam Cohort
Source: J Nutr Health Aging. 2023 Oct 31;27(11):1109–17. doi: 10.1007/s12603-023-2010-1 (PMC12876783; doi:10.1007/s12603-023-2010-1)
Supplement: Supplementary file 1 — Supplementary material, approximately 52 KB. [file mmc1.docx]

## Supplementary Table 1. Description of dietary patterns score calculation

| **Pattern score** | **Description** |
| --- | --- |
| EAT-Lancet Planetary Health Diet (EAT-L) | Based on dietary recommendations of the EAT-L report of 14 food groups based on 2500 kcal/day and were recalculated for 2000 kcal/day for women following a similar approach to Looman et al (1). Each recommendation was given a score between 0-10 points, calculated in men and women separately. Points were summed resulting in a score that ranged between 0 (no adherence) to 140 (complete adherence) points. Each dietary recommendation was categorized into adequacy, moderation, optimum or ratio scoring components as described by Colizzi et al (2) and adapted from Looman et al (1). Total energy intake was adjusted for in the analysis phase. |
| Traditional Mediterranean Diet Score (tMDS) adapted from Trichopoulou (3, 4) | Scores according to intake levels of cereals, fruits and nuts, vegetables, fish, legumes, dairy, meat based on tertiles (lowest 0 points, highest 2 points), olive oil intake: 0 for no consumption, 1 if below sex-specific median, 2 above, alcohol 2 points if between 5 and 25 g/d for women and 10 to 50 g/d for men, 0 if outside of range.  Total energy intake was adjusted for in the analysis phase. |

**Supplementary Table 2.**  Construction of EAT-L score, intake recommendations (g/day) and scoring for men based on 2500 kcal/day

| **Food Group** | **Component type^1^** | **HRD recommendation (g/day)** | **Minimum score**  **(0 points)** | **Proportional score** | **Maximum points**  **(10 points)** | **Proportional score** |
| --- | --- | --- | --- | --- | --- | --- |
| **Whole Grains^2^** |  |  |  |  |  |  |
| Rice, wheat, corn, and other | A | 464 | 0 g/d | 0-464 g/d | ≥464 g/d |  |
| **Vegetables** |  |  |  |  |  |  |
| All vegetables | A | 300 | 0 g/d | 0-300 g/d | ≥300 g/d |  |
| **Fruits** |  |  |  |  |  |  |
| All fruit^3^ | A | 200 | 0 g/d | 0-200 g/d | ≥200 g/d |  |
| **Tubers or starchy vegetables** |  |  |  |  |  |  |
| Potatoes and cassava | O | 50 | 0 g/d | 0-50 g/d | 50-100 g/d | 100-150 g/d |
| **Dairy foods** |  |  |  |  |  |  |
| Milk or derivative equivalents (e.g., cheese) | O | 250 | 0 g/d | 0-250 g/d | 250 – 500 g/d | 500-750 g/d |
| **Protein Sources** |  |  |  |  |  |  |
| Legumes | A | 50 | 0 g/d | 0-50 g/d | ≥50 g/d |  |
| Soy foods | A | 25 | 0 g/d | 0-25 g/d | ≥25 g/d |  |
| Beef, lamb and pork | M | 28 | ≥28 g/d | 28 -0 g/d | 0 g/d |  |
| Chicken and other poultry | O | 29 | 0 g/d | 0-29 g/d | 29-58 g/d | 58-88 g/d |
| Eggs | O | 13 | 0 g/d | 0-13 g/d | 13-25 g/d | 25-38 g/d |
| Fish | O | 28 | 0 g/d | 0-28 g/d | 28-100 g/d | 100-128 g/d |
| Nuts | O | 50 | 0 g/d | 0-50 g/d | 50-100 g/d | 100-150 g/d |
| **Added sugars** |  |  |  |  |  |  |
| All sweeteners | M | 31 | ≥31 g/d | 31-0 g/d | 0 g/d |  |
| **Added fats^4^** | R |  | No consumption of unsaturated fats OR ratio of unsaturated to saturated fats of ≤0.5 |  | No consumption of saturated fats OR ratio of unsaturated to saturated fats of ≥0.8 |  |

^1^A = adequacy component; O = optimum component; M = moderation component; R = ratio component. ^2^ Reference diet refers to dry, raw weight. Recommendations for whole grains were converted as described by Colizzi et al (2); ^3^ Excluding fruit juice. ; ^4^ Saturated fats included butter, lard and other animal fat; unsaturated fats included: vegetable oils and margarine

**Supplementary Table 3.** Construction of EAT-L score, intake recommendations (g/day) and scoring for women based on 2000 kcal/day

| **Food Group** | **Component type^1^** | **HRD recommendation (g/day)** | **Minimum score**  **(0 points)** | **Proportional score** | **Maximum points**  **(10 points)** | **Proportional score** |
| --- | --- | --- | --- | --- | --- | --- |
| **Whole Grains^2^** |  |  |  |  |  |  |
| Rice, wheat, corn, and other | A | 372 | 0 g/d | 0-372 g/d | ≥372 g/d |  |
| **Vegetables** |  |  |  |  |  |  |
| All vegetables | A | 240 | 0 g/d | 0-240 g/d | ≥240 g/d |  |
| **Fruits** |  |  |  |  |  |  |
| All fruit^3^ | A | 160 | 0 g/d | 0-160 g/d | ≥160 g/d |  |
| **Tubers or starchy vegetables** |  |  |  |  |  |  |
| Potatoes and cassava | O | 40 | 0 g/d | 0-40 g/d | 40-80 g/d | 80-120 g/d |
| **Dairy foods** |  |  |  |  |  |  |
| Milk or derivative equivalents (e.g., cheese) | O | 200 | 0 g/d | 0-200 g/d | 200 – 400 g/d | 400-600 g/d |
| **Protein Sources** |  |  |  |  |  |  |
| Legumes | A | 40 | 0 g/d | 0-40 g/d | ≥40 g/d |  |
| Soy foods | A | 20 | 0 g/d | 0-20 g/d | ≥20 g/d |  |
| Beef, lamb and pork | M | 23 | ≥23 g/d | 23-0 g/d | 0 g/d |  |
| Chicken and other poultry | O | 23 | 0 g/d | 0-23 g/d | 23-46 g/d | 46-69 g/d |
| Eggs | O | 10 | 0 g/d | 0-10 g/d | 10-20 g/d | 20-30 g/d |
| Fish | O | 22 | 0 g/d | 0-22 g/d | 22-80 g/d | 80-102 g/d |
| Nuts | O | 40 | 0 g/d | 0-40 g/d | 40-80 g/d | 80-120 g/d |
| **Added sugars** |  |  |  |  |  |  |
| All sweeteners | M | 25 | ≥25 g/d | 25-0 g/d | 0 g/d |  |
| **Added fats^4^** | R |  | No consumption of unsaturated fats OR ratio of unsaturated to saturated fats of ≤0.5 |  | No consumption of saturated fats OR ratio of unsaturated to saturated fats of ≥0.8 |  |

^1^A = adequacy component; O = optimum component; M = moderation component; R = ratio component.

^2^ Reference diet refers to dry, raw weight. Recommendations for whole grains were converted, as described by Colizzi et al (2)

^3^ Excluding fruit juice.

^4^ Saturated fats included butter, lard and other animal fat; unsaturated fats included: vegetable oils and margarine

**Note on scoring calculations**

For the adequacy components, the minimum score was given when there was no consumption of this component. Intake equal to the cut-off value or higher was given the maximum score of 10 points. The score for intake between zero and the cut-off value was calculated by dividing the reported intake by the cut-off value and subsequently multiplying the obtained ratio by 10. For the moderation components, 0 points were assigned if intake was above the threshold value. 10 points were assigned if intake was equal to or lower than the cut-off value. The score for intake between the threshold and cut-off values was calculated by dividing the difference between the intake and the cut-off value by the difference between the threshold value and the cut-off value. This ratio was subsequently multiplied by 10. The obtained score was subtracted from 10 to obtain the component score, as the score for moderation components has to decrease when intake increases. For the optimum component, the maximum score was assigned if intake was within the given range. No consumption was scored with the minimum score of 0 points. Intake lower than the cut-off value was scored by dividing the reported intake by the lower cut-off value of the range and subsequently multiplying the obtained ratio by 10. Intake between the higher cut-off value of the range and the threshold value was scored by dividing the difference between the intake and the cut-off value by the difference between the threshold value and the cut-off value. This ratio was subsequently multiplied by 10. The obtained score was subtracted from 10 to ensure that the score decreases when intake increases. For intake above the threshold value the minimum score of 0 points was given. Cut-off values and threshold values for the ratio components were set for the calculated ratios, instead of intakes. The maximum score of 10 points was assigned if the ratio was higher than the cut-off value. The minimum score of 0 points was assigned if the ratio was lower than the threshold value. The score for intake between the cut-off and threshold values was calculated by dividing the difference between the ratio and the threshold value by the difference between the cut-off and threshold value.

**Supplementary Table 4.** Associations between changes in dietary pattern score adherence over 6.8 year exposure (1994/1998 and 2001/2005) based on baseline median cut-off and long-term adjusted chemerin concentrations after 8.6 (average of 2 measurements from 2010/2012 and 2013)

|  | **Change in dietary pattern score from 1994/1998 and 2001/2005** | **Long-term chemerin (2010/2013)** | | |  |
| --- | --- | --- | --- | --- | --- |
|  |  | *% difference | SE | P value |  |
| **EAT-L** | Stable high vs stable low score | | | | |
|  | Model 1 | -4.86 | 2.59 | 0.052 |  |
|  | Model 2 | -4.43 | 2.63 | 0.081 |  |
|  | Increasing score vs stable low score | | | | |
|  | Model 1 | -4.50 | 2.67 | 0.081 |  |
|  | Model 2 | -3.96 | 2.72 | 0.133 |  |
|  | Decreasing score vs stable high score | | | | |
|  | Model 1 | -5.29 | 4.86 | 0.253 |  |
|  | Model 2 | -4.70 | 4.91 | 0.316 |  |
| **tMDS** | Stable high vs stable low score | | | | |
|  | Model 1 | -1.17 | 2.54 | 0.638 |  |
|  | Model 2 | -1.22 | 2.56 | 0.625 |  |
|  | Increasing score vs stable low score | | | | |
|  | Model 1 | -3.75 | 3.06 | 0.205 |  |
|  | Model 2 | -3.63 | 3.08 | 0.223 |  |
|  | Decreasing score vs stable high score | | | | |
|  | Model 1 | 2.58 | 2.44 | 0.291 |  |
|  | Model 2 | 2.71 | 2.46 | 0.270 |  |

Model 1 is adjusted for sex, age, baseline BMI, waist circumference, recreational sports, educational attainment, smoking status, prevalent diseases [hypertension (+ antihypertensive medication), type 2 diabetes, cancer (except non-melanoma skin cancer), CVD]), baseline chemerin, total energy intake at baseline and T1, and alcohol intake (EAT-L analysis).; Model 2 is additionally adjusted for available covariates at T2, including smoking status, recreational sports, alcohol intake (EAT-L analysis), and prevalent diseases [hypertension, type 2 diabetes, cancer, CVD]. Abbreviations: EAT-L, EAT-Lancet Planetary Health Diet; hs, high sensitivity; tMDS, traditional Mediterranean Diet Score; *% difference is calculated as (exp[linear regression coefficient] – 1)*100; a negative difference expresses the percentage reduction in biomarker concentration in the relevant exposure group compared to the reference group.

**Supplementary Table 5.** Associations between changes in dietary pattern score adherence over 6.8 year exposure (1994/1998 and 2001/2005) based on baseline upper quartile cut-off and long-term adjusted chemerin concentrations after 8.6 (average of 2 measurements from 2010/2012 and 2013)

|  | **Change in dietary pattern score from 1994/1998 and 2001/2005** | **Long-term chemerin (2010/2013)** | | |  |
| --- | --- | --- | --- | --- | --- |
|  |  | *% difference | SE | P value |  |
| **EAT-L** | Stable high vs stable low score | | | | |
|  |  | -1.21 | 2.63 | 0.639 |  |
|  | Increasing score vs stable low score | | | | |
|  |  | -3.27 | 2.20 | 0.126 |  |
|  | Decreasing score vs stable high score | | | | |
|  |  | -2.06 | 4.44 | 0.632 |  |
| **tMDS** | Stable high vs stable low score | | | | |
|  |  | 3.44 | 2.98 | 0.250 |  |
|  | Increasing score vs stable low score | | | | |
|  |  | -4.24 | 2.73 | 0.108 |  |
|  | Decreasing score vs stable high score | | | | |
|  |  | 0.12 | 3.57 | 0.973 |  |

Model is adjusted for sex, baseline age, baseline BMI, baseline waist circumference, baseline education, baseline chemerin, baseline and T2 recreational sports, baseline and T2 smoking status, baseline and T2 alcohol intake (EAT-L analysis), baseline and T2 prevalent diseases [hypertension (+ antihypertensive medication at baseline), type 2 diabetes, cancer (except non-melanoma skin cancer), CVD]), total energy intake at baseline and T1, and weight change categories (weight loss, weight maintenance, weight gain from baseline-T2)

Abbreviations: EAT-L, EAT-Lancet Planetary Health Diet; hs, high sensitivity; tMDS, traditional Mediterranean Diet Score; *% difference is calculated as (exp[linear regression coefficient] – 1)*100; a negative difference expresses the percentage reduction in biomarker concentration in the relevant exposure group compared to the reference group.

**Supplementary Table 6.** Long-term chemerin concentrations and proportion of high diet score adherence among weight change categories

|  | N (%) | Weight change in kg - Mean (SD) | Long-term chemerin – Median (IQR) | EAT-L diet score adherence Stable high - n (%) | tMDS score adherence – Stable high – n (%) |
| --- | --- | --- | --- | --- | --- |
| Weight loss (weight decrease >1kg) | 179 (27.4) | -5.3 (4.8) | 203.0 (171.2, 245.7) | 83 (48.3) | 72 (41.9) |
| Weight maintenance (weight change ≤ 1kg) | 92 (14.1) | 0.1 (0.6) | 197.0 (170.7, 238.6) | 40 (46.5) | 38 (44.2) |
| Weight gain (weight increase >1kg) | 382 (58.5) | 6.9 (4.7) | 213.8 (182.2, 246.5) | 168 (44.4) | 151 (40.0) |

Participants categorized into groups of weight loss, weight maintenance, and weight gain. Cut-points chosen based on those used in previous studies (5)

**Supplementary Table 7.** Associations between changes in dietary pattern score adherence and long-term adjusted biomarker concentrations additionally adjusted for weight loss, weight maintenance, and weight gain from T0-T2

|  | |  | | |
| --- | --- | --- | --- | --- |
| **Change in dietary pattern score from baseline to T1** | | **Long-term chemerin** | | |
|  | % difference | SE | P value |  |
| **EAT-L** | |  |  |  |
| Maintaining high score vs maintaining low score | |  |  |  |
|  | | -3.09 | 2.63 | 0.226 |
| Increasing score vs maintaining low score | |  |  |  |
|  | | -3.58 | 2.72 | 0.174 |
| Decreasing score vs maintaining high score | |  |  |  |
|  | | -6.22 | 4.95 | 0.184 |
| **tMDS** | |  |  |  |
| Maintaining high score vs maintaining low score | |  |  |  |
|  | | -0.18 | 2.54 | 0.940 |
| Increasing score vs maintaining low score | |  |  |  |
|  | | -2.26 | 3.08 | 0.451 |
| Decreasing score vs maintaining high score | |  |  |  |
|  | | 2.48 | 2.43 | 0.308 |

Model is adjusted for sex, baseline age, baseline BMI, baseline waist circumference, baseline education, baseline chemerin, baseline and T2 recreational sports, baseline and T2 smoking status, baseline and T2 alcohol intake (EAT-L analysis), baseline and T2 prevalent diseases [hypertension (+ antihypertensive medication at baseline), type 2 diabetes, cancer (except non-melanoma skin cancer), CVD]), total energy intake at baseline and T1, and weight change categories (weight loss, weight maintenance, weight gain from baseline-T2)

**References to Supplemental Material**

1. Looman M, Feskens EJ, de Rijk M, Meijboom S, Biesbroek S, Temme EH, et al. Development and evaluation of the Dutch Healthy Diet index 2015. Public Health Nutr. 2017;20(13):2289-99.

2. Colizzi C, Harbers MC, Vellinga RE, Verschuren WM, Boer JM, Temme EH, et al. Adherence to the EAT-Lancet Healthy Reference Diet in relation to Coronary Heart Disease, All-Cause Mortality Risk and Environmental Impact: Results from the EPIC-NL Cohort. medRxiv. 2021:2021.06.30.21259766.

3. Trichopoulou A, Costacou T, Bamia C, Trichopoulos D. Adherence to a Mediterranean diet and survival in a Greek population. The New England journal of medicine. 2003;348.

4. Galbete C, Kröger J, Jannasch F, Iqbal K, Schwingshackl L, Schwedhelm C, et al. Nordic diet, Mediterranean diet, and the risk of chronic diseases: the EPIC-Potsdam study. BMC Medicine. 2018;16(1):99.

5. Paige E, Korda RJ, Banks E, Rodgers B. How weight change is modelled in population studies can affect research findings: empirical results from a large-scale cohort study. BMJ Open. 2014;4(6):e004860.
